# Thermoelectric Properties of Tin Telluride Quasi Crystal Grown by Vertical Bridgman Method

**DOI:** 10.3390/ma12183001

**Published:** 2019-09-16

**Authors:** Yue-Xing Chen, Fu Li, Delong Li, Zhuanghao Zheng, Jingting Luo, Ping Fan

**Affiliations:** 1 Shenzhen Key Laboratory of Advanced Thin Films and Applications, College of Physics and Optoelectronic Engineering, Shenzhen University, Shenzhen 518060, China; chenyx@szu.edu.cn (Y.-X.C.); lifu@szu.edu.cn (F.L.); zhengzh@szu.edu.cn (Z.Z.); luojt@szu.edu.cn (J.L.); 2 College of chemistry and environmental engineering, Shenzhen University, Shenzhen 518060, China

**Keywords:** thermoelectric, tin telluride, quasi crystal, transport properties

## Abstract

Tin telluride (SnTe), with the same rock salt structure and similar band structure of PbTe alloys, was developed as a good thermoelectric material. In this work, SnTe quasi crystal was grown by vertical Bridgman method, with texturing degree achieved at 0.98. Two sets of samples, perpendicular and parallel to the growth direction, were cut to investigate thermoelectric properties. As a result, a carrier concentration (*p*_H_) of ~9.5 × 10^20^ cm^−3^ was obtained, which may have originated from fully generated Sn vacancies during the long term crystal growth. The relatively high Seebeck coefficient of ~30 *μ*VK^−1^ and ~40 *μ*VK^−1^ along the two directions was higher than most pristine SnTe reported in the literature, which leads to the room temperature (*PF*) for SnTe_IP and SnTe_OP achieved at ~14.0 *μ*Wcm^−1^K^−2^ and ~7.0 *μ*Wcm^−1^K^−2^, respectively. Finally, the maximum dimensionless figure of merit (*ZT*) values were around 0.55 at 873 K.

## 1. Introduction

Thermoelectric material is considered a solution for sustainable development in the future, as it can realize the direct conversion of waste heat into useful electricity. The efficiency of thermoelectric materials is determined by the dimensionless figure of merit (*ZT*), which is defined as:

*ZT* = *S*^2^*σT*/(*κ*_el_ + *κ*_L_)

where *S*, *σ*, *κ*_el_, *κ*_L_ and *T* are the Seebeck coefficient, electrical conductivity, electronic thermal conductivity, lattice thermal conductivity and absolute temperature, respectively [1,2,3]. To achieve high *ZT*, materials should possess a large Seebeck coefficient, high electrical conductivity and low thermal conductivity, simultaneously. Tin telluride (SnTe) has the same rock salt structure and similar band structure as traditional PbTe alloys. The PbTe based compounds perform best with thermoelectric properties in a medium temperature range [4,5,6,7,8]. More recently, because of the environmental concern over toxic lead elements, lead-free SnTe alloys have received lots of attention [9,10,11,12,13,14,15,16,17,18]. Many researchers are trying to optimize carrier concentration by band structure engineering with element doping and improved thermoelectric properties [9,10,11,12,13,17,18]. This strategy, for example, has been performed well in functionally graded materials (FGMs), such as thermoelectric materials [19,20]. As for the SnTe compounds, indium doping prompts the resonant states in the valence bands and causes the significant enhancement of the Seebeck coefficient, which results in enhancement of maximum *ZT* from 0.8 for a pristine sample to 1.1 at 873 K [13]. In addition, a combination of band convergence and interstitial defects has led to a peak high *ZT* of 1.6 at 900 K in the complex chemical constitution of Sn_0.91_Mn_0.14_Te(Cu_2_Te)_0.05_, where *ZT* is higher than any other SnTe based compounds [18]. Pristine SnTe usually has a carrier concentration (*p*) magnitude of ~10^20^ cm^−3^ which, due to the intrinsic Sn vacancies, leads to low *S* and high *κ*_el_. It has been reported that SnTe has a large valence band offset of about 0.3–0.4 *e*V between light and heavy valence bands at room temperature, which gives rise to a unique Pisarenko relation [21,22]. In the Pisarenko plot, the minimum Seebeck coefficient is obtained when *p* = 1~2 × 10^20^ cm^−3^, and the maximum is achieved at about 8 × 10^20^ cm^−3^, which means pristine SnTe at high carrier concentration levels may also lead to good thermoelectric performance. Pristine SnTe prepared by different methods usually results in low *S*, as the *p* is located in the range of 1.0 to 5.0 × 10^20^ cm^−3^, and poor *ZTs* at room temperature [12,17,18,21,22]. In the reported literature, pristine SnTe prepared by zone-melting methods possessed large hole carrier concentration due to fully generated Sn vacancies [12]. The researchers reduced the hole carriers by valence band engineering with Mn doping and optimized the thermoelectric properties. However, as mentioned above, SnTe with high *p* may also be of benefit to the enhancement of thermoelectric performance. Hence, in this work, we grew SnTe quasic crystal using the vertical Bridgman method, as grown crystal possesses high carrier concentration levels of ~9.5 × 10^20^ cm^−3^ and relatively high Seebeck coefficient of ~30 *μ*VK^−1^. We also investigated the thermoelectric properties of grown SnTe quasic crystal perpendicular and parallel to the growth direction, respectively. This work presents a new insight into further understanding the electrical transport properties of pristine SnTe. 

## 2. Experimental 

SnTe ingot was first synthesized by conversional solid solution method. High purity raw materials, Sn shots (99.99%, metals basis, Alfa Aesar, Heysham, UK) and Te lumps (99.999%, metals basis, Aladdin, Shanghai, China), were used. Stoichiometric proportions of the constituent elements, tin and tellurium, were weighed and put into a silica tube with an inner diameter of 12.5 mm, the tube was then evacuated to a pressure of ~10^−4^ Pa before being flame sealed. The silica tube was steadily heated to 673 K over 4 h, maintained for 4 h, then heated to 1073 K over 4 h, and maintained for a further 12 h, followed by furnace cooling. The obtained SnTe ingot was taken out, crushed and reloaded in a customized silica tube with a cone shape bottom and an inner diameter of *Φ* 12.5 mm. The tube was evacuated and sealed again, and then put into a modified vertical Bridgman furnace, in which the temperature at the upper heating part was 100 K higher than that of the bottom cooling part. The upper heating temperature was set at 1123 K, and the lowering rate of the tube during the growth process was set at 1.5 mmh^−1^. The grown crystal was cut into pieces for the purpose of thermoelectric measurement and characterization. Two sets of specimens, perpendicular and parallel to the growth direction, were cut for electrical and thermal property measurement. In this work, we defined the specimens as in plane (SnTe_IP) and out of plane (SnTe_OP) directions, respectively. Phase structures were detected by x-ray diffraction (XRD, CuKα, Riguku, Tokyo, Japan). The morphologies of fractographs were investigated by field emission scanning electron microscopy (FESEM, Zeiss Merlin, Oberkochen, Germany), HAADF (High Angle Annular Dark Field), and SAED (Selected Area Electron Diffraction). HAADF-STEM (High Angle Annular Dark Field- Scanning Transmission Electron Microscope) images were measured using a transmission electron microscope (Titan Cubed Themis G2 300, FEI, Waltham, MA, USA).

The *S* and *σ* were measured from 300 to 900 K in a thin helium atmosphere using an ZEM-3 instrument, Ulvac-Riko, Yokohama, Japan. The *κ* was calculated using the relationship of *κ* = *DCpd* with density (d) measured using the Archimedes method, and the thermal diffusivity (D) measured using laser flash equipment ( Laser Flash Apparatus LFA 457, NETZSCH, Bavaria, Germany). The specific heat (*Cp*) was calculated from the *C*_p_ (*k*_B_/atom) = (3.07 + 0.00047(*T*/K-300)), which was determined from the measured values of Blachnik and lgel [23]. The carrier concentration (*p*_H_) was derived from the formula *p*_H_ = 1/*e*R_H_, where *e* is the electronic charge and R_H_ is the Hall coefficient, obtained under a reversible magnetic field (0.8 *T*) by the Van der Pauw method using a Hall measurement system (Lake Shore 8400 Series, Model 8404, Lake Shore, Westerville, OH, USA) in a wide temperature range from 300 to 873 K. The mobility (*μ*_H_) was calculated by *μ*_H_ = *σ*R_H_. 

The uncertainty of the Seebeck coefficient and electrical conductivity measurements were within 5%, and thermal conductivity was estimated to be within 8% when considering the uncertainties for D, Cp and d. Therefore, the combined uncertainty for all measurements involved in the *ZT* determination, as shown in the plot, was estimated to be 15–20%.

## 3. Results and Discussion

**Figure 1:**
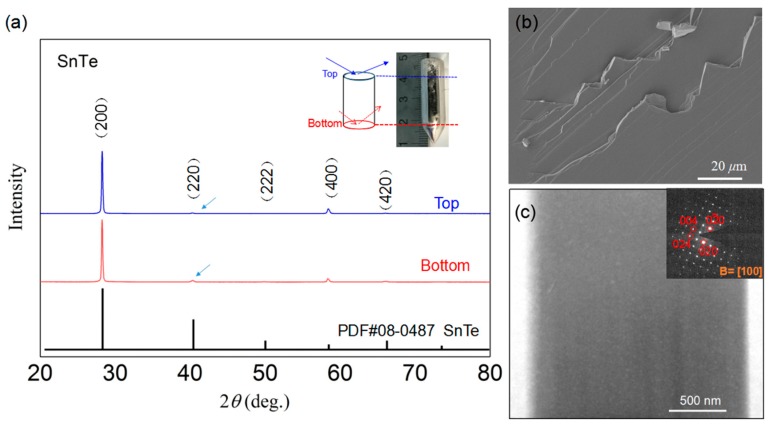
(**a**) XRD patterns of SnTe quasi crystal for both bottom and top planes, perpendicular to the growing direction. The inset figure shows a picture of the sample and a schematic diagram of measurement directions; (**b**) field emission scanning electron microscopy (FESEM) images for fracture surface structure of SnTe quasi crystal; (**c**) [100] zone-axis low magnification TEM image with inserted fast Fourier transformation (FFT) patterns.

Figure 1a shows the XRD patterns for SnTe quasi crystal on both the bottom and top parts perpendicular to the growth direction (schematic diagram shown in the inset figure). The inset picture also shows the appearance of the crystal, which had a bright and metallic luster, with measurements of 35 mm in length and 12.5 mm in diameter. As shown in Figure 1a, the XRD patterns were well-marked with standard card PDF#08-0487 of SnTe (Fm-3m space group, *a* = *b* = *c* = 6.328 Å), which indicates the formation of the single phase for the whole crystal. It is worth noting that a very strong orientation along the (*h*00) directions was observed, while other planes were hardly noticed. This indicates that the grown sample could be quasi crystal. We further estimated the texturing degree (*F*) by using the Lotgering method, with the following typical formula:

*F*(*hkl*) = (*P* − *P*_0_)/(1 − *P*_0_)

where *P* and *P*_0_ are the ratios of the integrated intensities of all (*hk0*) crystal planes to those of all (*hkl*) planes for preferentially and randomly oriented samples, respectively [24,25]. In this work, *P* is the ratio of integrated intensities for the (*h*00) plane and overall (*hkl*) planes based on the measured XRD patterns, and *P*_0_ is calculated from the standard card data with the same processing. As a result, the texturing degree *F*(*h*00) achieved was 0.98, which was close to unity. As we already know, the *P* will be unity for a single crystal, and the corresponding *F* will be unity too. Therefore, the sample obtained in this work can be considered quasi crystal with very high orientations. The FESEM image (Figure 1b) of fracture surface structures shows that no grain boundaries, pores or impurities were observed. In addition, the low magnification TEM images, with inserted fast Fourier transformation (FFT) patterns (Figure 1c), show the single crystal characteristics in the full field of observation. The results further confirm the good quality of the quasi crystal of SnTe. 

**Figure 2:**
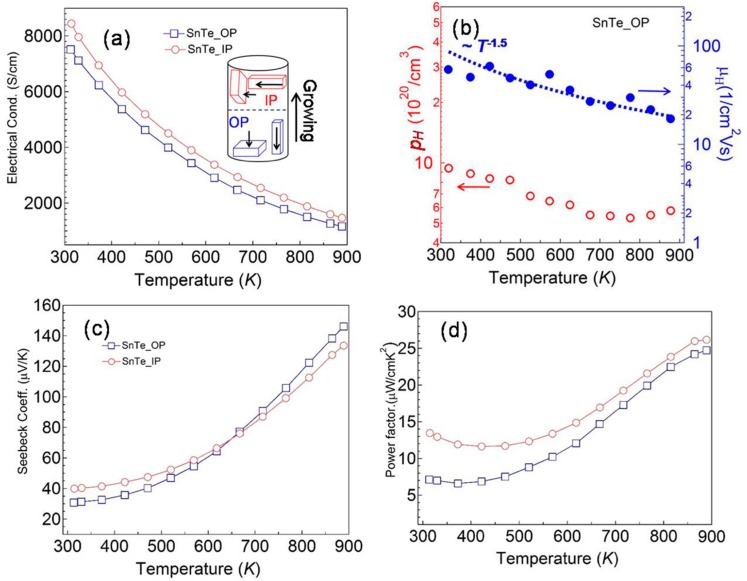
Temperature dependence of electrical transport properties (**a**) electrical conductivity; (**b**) carrier concentration and mobility; (**c**) Seebeck coefficient; (**d**) power factor for SnTe quasi crystal. The inset figure in (a) shows the schematic diagram of measuring directions.

Figure 2 shows the electrical transport properties for grown quasi crystal. In this work, we measured the thermoelectric properties along two directions (SnTe_OP and SnTe_IP) as displayed in the inset of Figure 2a. Electrical conductivities (*σ*), as a function of temperature from 300 to 900 K for SnTe_IP and SnTe_OP, respectively, are displayed in Figure 2a. For two *σ*(*T*) curves, the electrical conductivity decreased as the temperature increased, which indicates a typical behavior of a degenerated semiconductor. This heavily doped behavior is normally observed in other pristine and element doped SnTe samples [12,13,14,15,16,17]. In such samples, the natural generated Sn vacancies led to a high carrier concentration. The *σ* for the SnTe_IP samples was over 8,000 Scm^−1^ at 300 K and then decreased; the value was higher than that of the SnTe_OP in the whole temperature range. This difference is understandable in such a quasi crystal, as the cutting direction for the SnTe_IP sample would not have been the same as that of the SnTe_OP sample because it was cut along the growth direction. We performed the XRD measurement on the SnTe_IP sample; the result showed the preferred orientation along the (420) plane. The differences could also be found on the *S*(*T*) curve, which requires further excavating with theoretical calculations. The Hall measurement was applied on the SnTe_OP sample in order to obtain the variation in *σ* (*T*); the results are plotted in Figure 2b. Normally, *σ* is proportional to the carrier concentration (*p*_H_) and carrier mobility (*μ*_H_) as *σ* = *p*_H_*μ*_H_*e*, where *e* is the electron charge. As shown in Figure 2a, the *p*_H_ was about 9.5 × 10^20^ cm^−3^ at 300 K, with the value gradually decreasing to about 5.0 × 10^20^ cm^−3^ above 700 K. The *p*_H_ at 300 K was the highest for all reported SnTe based samples, which may have been due to the fully generated Sn vacancies during the long term growing process. As indicated by the variation in *μ*_H_, the value decreased as the temperature increased and followed the *T*^−1.5^ law which indicates the hole carriers were majorly scattered by long-wave acoustic phonons. Therefore, the reduction in both *p*_H_ and *μ*_H_ contributes to the decrease in *σ*. The *S*(*T*) for SnTe_IP and SnTe_OP (Figure 2c) increased with the temperature, which was consistent with the temperature dependency in *σ*(*T*) and the hall measurement results. The room temperature (*S*) for SnTe_IP and SnTe_OP was ~40 *μ*VK^−1^ and ~30 *μ*VK^−1^, respectively, which was higher than most pristine SnTe in the literature, and comparable with SnTe alloys prepared by the zone melting method [12]. This may be due to the Fermi level crossing the band offset and locating deeply in the valence bands, which then leads to two band contributions. Hence, the calculation of the power factor (*PF*) = *S*^2^*σ* is displayed in Figure 2d. The room temperature (*PF*) for SnTe_IP and SnTe_OP was ~14.0 *μ*Wcm^−1^K^−2^ and ~7.0 *μ*Wcm^−1^K^−2^, respectively, with values enhanced significantly to ~26.5 and 25.0 *μ*Wcm^−1^K^−2^ at 900 K. 

**Figure 3:**
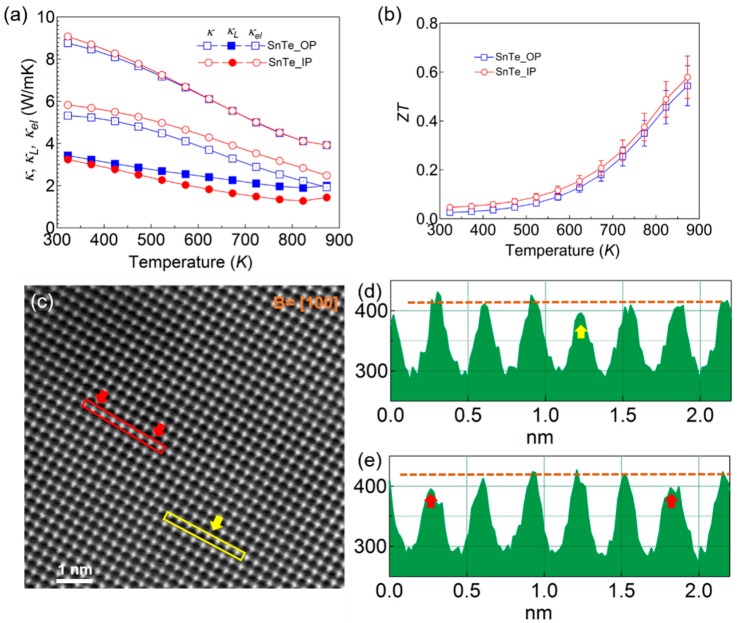
Temperature dependence of thermal transport properties (**a**) and *ZT* with error bars; (**b**) for SnTe quasi crystal; (**c**) typical HAADF-STEM image viewing along [100] zone axis. Intensity profile of the square root of STEM intensity from (**d**) yellow box area; (**e**) red box area, respectively.

The total thermal conductivity (*κ*), electronic contribution (*κ*_el_) (*κ*_el_ = *LTσ*, where *L* is the Lorenz number estimated from a single Kane band model) and lattice thermal conductivity (*κ*_L_) (*κ*_L_ = *κ − κ*_el_) are all shown in Figure 3a. There were slight differences observed in the two *κ*(*T*) curves, where the values were ~9.0 Wm^−1^K^−1^ at 300 K decreasing to 3.9 Wm^−1^K^−1^ at 873 K, higher than that of other polycrystalline SnTe samples [12,13,14,15,16,17,18]; this was mostly due to the impressive *κ*_el_ part, which made up almost two-thirds of the *κ*(*T*). The lattice contributions (*κ*_L_) decreased with the temperature, roughly following the variation of *T*^−1^ which indicates that the dominant scattering factor was phonon-phonon scattering. This value is not surprising at room temperature, as the grain boundary scattering is negligible in such a quasi crystal. The maximum *ZT* values along the two directions were around 0.55 at 873 K, comparable with those previously reported [12,13,14,15].

Figure 3c shows the typical HAADF-STEM image viewing along the [100] zone axis. The atoms were arranged according to the crystal structure of SnTe. We also obtained the intensity profile, which was proportional to the atomic number Z from the yellow and red box areas (Figure 3d). The yellow and red arrows identify the columns where intensities were lower than that of adjacent columns. This may indicate the lack of atoms for the corresponding columns, and provides indirect proof of the existence of abundant Sn vacancies. 

**Figure 4:**
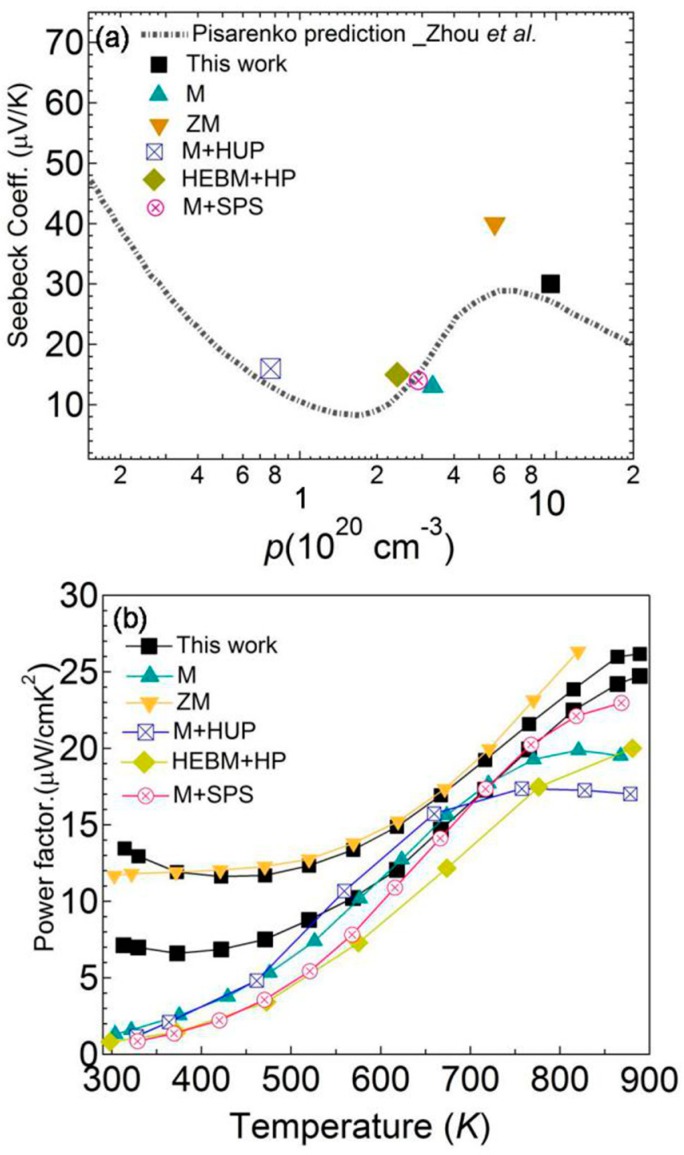
(**a**) Room temperature (*S*) as a function of *p*_H_; (**b**) *PF*s as a function of temperature for pristine SnTe prepared by different methods. The dotted line is a Pisarenko line calculated by a two valence band model. For comparison, experimental data of pristine SnTe prepared by melting (M) [14], zone melting (ZM) [12], melting followed by hot uniaxial pressing (M+HUP) [15], high-energy ball mill followed by hot press (HEBM+HP) [13], and melting followed by spark plasma sintering (M+SPS) [17] are also given.

We plotted the room temperature (*S*) as a function of *p*_H_ with the Pisarenko line (dotted line in Figure 4a) of SnTe, which was calculated using a two valence band model by Zhou et al. The experimental data for pristine SnTe, prepared by melting (M) [14], zone melting (ZM) [12], melting followed by hot uniaxial pressing (M+HUP) [15], high-energy ball mill followed by hot press (HEBM+HP) [13], and melting followed by spark plasma sintering (M+SPS) [17], as well as the sample in this work, were plotted for comparison. It can been seen that the *S* for samples, prepared by using HP or SPS as a final compacted process, usually has a carrier concentration of around 1.0 to 3.0 × 10^20^ cm^−3^. The experimental data located in the Pisarenko line demonstrates the strength of the adopted physical model. On the other hand, due to the long term growing process, samples prepared by zone melting or vertical Bridgman method have carrier concentrations larger than 5.0 × 10^20^ cm^−3^ and relatively high *S,* as shown in Figure 4a. Our results strengthen the practicability of this calculation model, and suggest that the contribution of two valence bands leads to a high *S*. As a result, the room temperature (*PF*s) was much higher than other samples with lower *p*_H_, as shown in Figure 4b. Higher values were maintained across the whole temperature range, which is beneficial in improving average *ZT* values. The results in this work are instructive to purchase better thermoelectric performance on SnTe alloys. 

## 4. Conclusions

SnTe quasi crystal, with measurements of 35 mm in length and 12.5 mm in diameter, was grown using the vertical Bridgman method. The good quality of the crystal was confirmed by XRD and low magnification TEM results. The texturing degree of 0.98 was calculated based on the XRD patterns. The FESEM images showed few grain boundaries, as observed in the fracture surfaces. Thermoelectric properties for the two sets of samples, perpendicular and parallel to the growth direction, were investigated. High carrier concentration (*p*_H_) ~9.5 × 10^20^ cm^−3^ originates from fully generated Sn vacancies during the long term crystal growth process. The room temperatures (*S*) of ~30 *μ*VK^−1^ and ~40 *μ*VK^−1^ along the two directions, respectively, were higher than most of the reported pristine SnTe samples, which may be due to the Fermi level crossed by the band offset and located deeply in the valence bands, which then leads to two band contributions. As a result, the room temperature (*PF*) for SnTe_IP and SnTe_OP was achieved at ~14.0 *μ*Wcm^−1^K^−2^ and ~7.0 *μ*Wcm^−1^K^−2^, respectively, and the maximum *ZT* values of 0.55 at 873 K were achieved along the two directions. Compared with other pristine SnTe prepared by different methods, the sample grown in this study showed high carrier concentration (*p*_H_) and room temperature (*PF*), which is beneficial in improving average *ZT* values.
